# Mandatory verses voluntary self-tests for new online casino customers: effect on engagement, quality, gambling behavior and use of responsible gambling measures

**DOI:** 10.1186/s12954-025-01173-4

**Published:** 2025-02-20

**Authors:** Jakob Jonsson, Nathan Lakew, Philip Lindner

**Affiliations:** Centre for Psychiatry Research, Department of Clinical Neuroscience, Karolinska Institutet & Stockholm Health Care Services, Region Stockholm, Norra Stationsgatan 69, 113 64 Stockholm, Sweden

**Keywords:** Self-test, Mandatory Duty-of-care, Responsible gambling, Prevention

## Abstract

**Background:**

To combat the public health concern that is problem gambling, gambling operators are increasingly being required by legislation to exercise a duty of care obligation, including the provision of Responsible Gambling (RG) tools. Self-test assessments have long been a popular RG tool implemented by many operators, yet there has been scant empirical research on self-tests, including on how the method of delivery impacts engagement, quality, and subsequent gambling behavior. The main objective of the current study was to examine if the level of voluntariness to perform a self-test moderated these key outcomes.

**Method:**

Participants in the study, 1800 new online customers at a leading Swedish gambling company, were randomized to one of three arms: No message (control), up to four messages inviting them to do a self-test, and one message with a mandatory (but technically possible to circumvent) self-test. The interventions were presented when the customer logged in.

**Results:**

The results showed that 38.9% in the mandatory group and 4.8% in the voluntary group completed the self-test, with indications of a somewhat lower quality of the test by the mandatory group. There was no difference in customer churn or gambling behavior, and only minor differences in use of RG-measures post intervention.

**Conclusions:**

We conclude that presentation format matters and can affect the use and quality of tests: gambling operators should exercise caution when interpreting non-risk assessment results derived solely from self-test tools, particularly mandatory ones, as it can result in inaccurate risk assessments that may mislead duty of care obligations. The balance between achieving high participation and maintaining quality (and thereby meaningfulness) is discussed.

## Introduction

There is an increasing demand for gambling operators to implement duty-of-care measures to alleviate problem gambling [1]. In a recent comprehensive review, for example, Meerkerit [2] reported that eleven European countries currently have duty of care regulations in their legislation. Duty of care issues include providing a means for monitoring one’s gambling consumption, access to RG and self-exclusion possibilities, and making contact with customers as well as taking action to limit gambling activities when warranted [3, 4]. Other duty of care measures such as affordability checks, education awareness programs, and channeling problem gamblers to healthcare systems were explored from both policy and operators’ perspectives to minimize gambling harms [5–7].

Given their ease of use and cross-purpose applications, self-test tools have been popular among operators as part of fulfilling their duty of care [8]. These tools provide a means to evaluate one’s gambling to determine if there is a presence of harm in terms of behavior, attitude as well as time spent in gambling activities. In addition, research shows that confidentiality, conveniences in terms of when to complete an assessment, and fast feedback loops have made self-test tools practical in both population-based research and clinical settings [9, 10, 11]. As such, there has been ample research work in the field that focuses on developing and testing screening self-test tools across different contexts with both preventive and intervention approaches in mind [12–14].

Despite wide interest in the use of self-test tools as a duty of care measure, there is still scant research examining how the method of delivery affects their adoption, quality of performed tests, and outcome on gambling behavior. The main objective of the current study is to examine if the level of voluntariness of self-tests affects the uptake and effects. To this end, we compare two self-test delivery methods—mandatory and voluntary action pop-ups—and analyze the quality of performed tests in terms of time spent to complete the assessment, response quality, and their effect on future gambling behavior and use of responsible gambling tools. In addition, we will evaluate if the different presentation of self-tests affects customer retention and continuity of using gambling services.

## Self-test: approach, setting, and format of implementation

The discussion of implementing self-tests in contrasting forms as mandatory or volunteer measures can be appreciated in the context of the field’s ongoing debate on how to problematize and tackle harmful gambling [15]. On one hand, there is a general consensus within the public health approach to conceptualize problem gambling not only as a treatable condition but also as a preventable one [16, 17]. In such framing, more stringent and mandatory measures are seen as an effective prevention approach, albeit treatment and intervention remain a major effort [18]. As such, in the context of public health approaches, self-test tools mainly fit into an early, at times obligatory, screening mechanism with a binding intervention if the risk is indicated [8].

On the other hand, we have the Reno model which has significantly influenced the majority of RG measures introduced by gambling operators, emphasizing educational guidelines and voluntary tools as the most effective approaches to RG, rather than normative measures focused on harm prevention [18–20]. In such framing, personal choices (hence responsibilities) are considered the main problem/solution space. In the same line of vein, the Reno model stipulates that different stakeholders may bear responsibilities to minimize gambling problems, however, individuals are ultimately responsible for making informed decisions [21]. Consequently, RG measures including self-test tools’ main role focus on facilitating informed choices, where interventions are reserved for validated risk behavior.

The effectiveness of both mandatory and voluntary self-assessment formats depends heavily on an individual’s willingness and honest participation. Without external enforcement, such as care supervision, these assessments can be undermined by factors such as a lack of self-motivation, denial and avoidance, or biases in evaluating one’s behavior. Consequently, the approach can subtly emphasize high levels of personal motivation and active involvement, which naturally encourage the development of self-responsibility. As such, the use of self-assessment tools within the context of RG can be viewed as supporting the broader goal of fostering autonomy and accountability toward one’s gambling activities. That is, on a deeper philosophical level, the reliance of voluntary self-assessment formats on personal engagement aligns closely with the principles of the Reno approach.

In addition to the above two approaches, current research suggests self-test tools typically employed in two main settings—clinical and population-based works—as a diagnosis method of disordered gambling, a selection mechanism to identify relevant research samples, or as RG measure among operators [10, 22, 23]. In clinical settings, self-test tools are mainly used as a screening tool to recommend and/or perform further clinical interventions, and tend to be voluntary [24, 25]. Self-tests in population settings were also applied to assess gambling-related harm on a bigger population scale such as regional [26, 27] or national level [28]. In both cases of population-oriented research, self-test tools provide a convenient and quick way to gauge problem gambling, however, their operationalization tends to be, again, voluntary with scant follow-up research on their effect of use.

Under the umbrella of population-oriented implementation, self-test tools are widely integrated within the duty of care strategies of gambling companies. Self-test tools enabled operators to combine both prevention and intervention strategies in the form of screening, treatment referrals, and as part of their behavioral tracking mechanisms [23, 29].

Hence, current research indicates that self-test tools tick many of the duty of care checkboxes for both intervention and prevention approaches as well as for clinical and population operational settings. Despite their widespread use, uncertainty persists regarding how to maximize participation and compliance with self-test tools, which may impact their effectiveness as a duty-of-care measure. Some indications show the effect of delivery format on the quality of RG measures. For example, a previous finding [30] shows that around two-thirds of Swedish poker players were compelled to set a reasonable monetary limit when it was mandatory to do so. Such a result implies that the outcome of RG tools could be affected depending on the mandate of compliance (i.e., enforced or optional), however, there exists scant scientific research in validating such a claim in a self-test setting. Given the field’s ambition of promoting both preventive and intervention-based RG measures across operators, it is important to understand the effect of mandatory self-tests on the quality of responses and retention effect on customers. Finally, the field can benefit from comprehensive insight into the impact of mandatory vs. voluntary self-assessment tools promotion on gambling platforms as a duty of care measure [31].

## Current study

The overall ambition of the current study is to investigate the use and effects of gambling self-tests as an RG measure. Specifically, we investigate how modes of an invitation to the self-test influence the tendency to engage with the test and the subsequent effects on gambling behavior. We compare a more mandatory condition (technically possible to circumvent) with receiving multiple reminders and a non-intervention control group. We will also look at the effect on future gambling behavior, and players’ tendency to use other RG tools.

## Objective and hypotheses

This study investigates two different methods of presenting the self-test to examine what effects it has on self-test performance, gambling behavior, and use of other RG tools. In doing so, the study compared mandatory and voluntary gambling self-test delivery methods with a control group., The voluntary approach involves up to four messages inviting customers to complete a self-test. We hypothesized that:

### H1

The group receiving the mandatory self-test will perform more self-test than the other groups.

### H2

The group receiving the mandatory self-test will have a lower quality of their self-test (time on test, response patterns) compared to the soft group.

### H3

The mandatory group will subsequently gamble less in terms of time, bets, net losses, and number of days compared to the soft group & controls, and the soft group will gamble less than the controls.

### H4

The mandatory group will use more RG tools than the other groups.

### H5

There will be no difference in “customer survival” (people staying as customers) between the groups four weeks after intervention.

## Method

### Setting

The study was conducted at Svenska Spel Sport & Casino, a Swedish state-owned gambling company with on- and off-line sport and horse betting, online casino, poker, and bingo. All gambling at the company is identified and registered to the individual gambler in the company’s data warehouse.

### Participants

The sample comprised 1825 new customers at Svenska Spel who registered an account 28–60 days prior to the study. To be included, customers had to have engaged in online casino gambling on at least three different days, including at least one day in the week preceding their inclusion.

### Intervention procedure

**Fig. 1:**
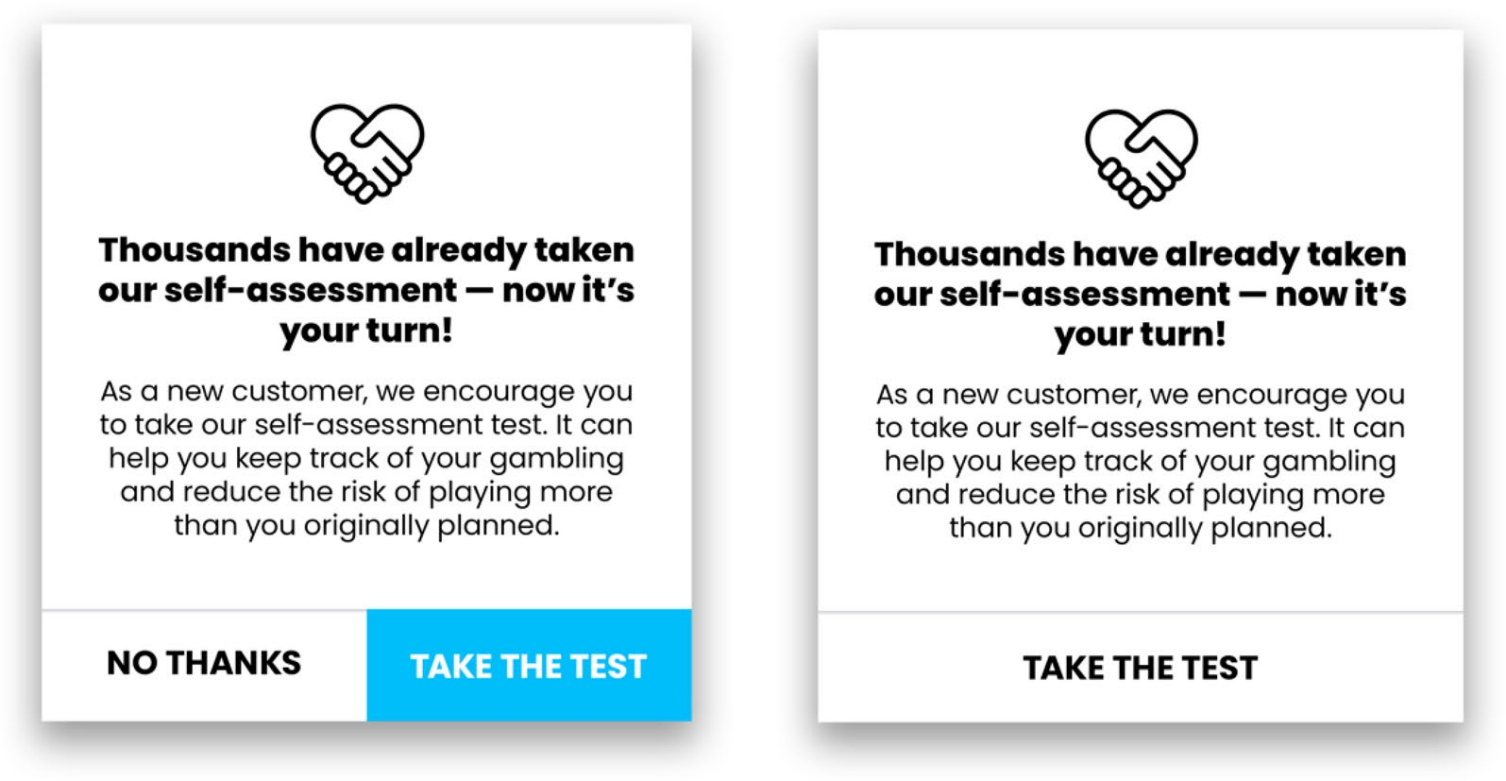
Invitation showed on screen to take a self-test

Participants were randomized to one of three arms: No message (control), up to four messages inviting them to do a self-test (non-mandatory – “soft” messages), and one message with a mandatory self-test, see Fig. 1. The interventions (soft messages and mandatory self-test) were presented when the customer logged in. For the mandatory self-test, the customer needed to take the self-test before they got access to the gambling opportunities; it was however technically possible to circumvent it by clicking on the link and then go back again using the back navigating arrow button in the web browser. GamTest [29] was used as the online self-test. It consists of 15 statements with a response scale 0–10 and it gives feedback and recommendations based on the customers’ answers. GamTest has shown high correspondence with the players’ own understanding of their problems and with PGSI. It captures five dimensions of problematic gambling (i.e., overconsumption of money and time, and monetary, social and emotional negative consequences) with high reliability.

### Measures and data collection

Data collection was between July 2 and September 9th 2024. Account-level gambling data and responsible gambling (RG) data were compiled and collapsed into either four weeks before (pre) or four weeks (post) after intervention. Date for intervention for the voluntary group was set to when the self-test was performed after invitation, and if no test performed to the day of the last reminder. Date set for the control group was the same as for the mandatory group.

The following gambling measures were compiled from Svenska Spel’s data warehouse: the number of days played, net losses, theoretical loss (bet × 1-payback percentage), and time spent gambling. The following RG measures were collected from the data warehouse: the number of increased limits, the number of lowered limits, the monthly deposit limit on day 7 of each week, the number of self-exclusions, the number of visits to “My Gambling Habits” (an RG hub with gambling feedback, limits, and recommendations), user behavior (clicks) during visits “My Gambling Habits,”. We received data on the start and completion of self-tests, result on item-level and the test time.

Quality indicators for low quality of the self-test was defined as using less than 3 s per statement or scoring 0 on all items or 10 on all items.

### Statistical analysis

Data were analyzed in SPSS, Version 28. For hypotheses H1, H2, H4, and H5, the Chi-square test was used. To test the effect differences for hypothesis H3, four Repeated measures GLM were applied looking at time * group interaction for theoretical loss, net expenditure, days played and time spent respectively. Groups were the conditions: control, voluntary and mandatory. As we performed multiple statistical analyses to test H3, we set our alpha level at 0.0125 to reduce the Type-I error rate for the GLM.

## Results

The results indicated that, in the mandatory condition, 43.0% started the test, and 38.9% completed it. In the voluntary condition, 5.5% started the test, and 4.8% completed it. In the control group, 0.3% started and finished the test. The Chi-square values for the ‘started’ and ‘finished’ measures were as follows: (2, 1825) = 484, *p* < 0.001, and (2, 1825) = 431, p < 0.001. Of the n = 265 completed self-tests among gamblers in the intervention groups, 86.4% were non-problematic, 9.1% at risk, and 4.5% problematic, which corresponds to 2% at risk and 1% problematic of all invited gamblers, Additionally, a higher proportion of participants in the mandatory group (34%) spent less than 3 s per self-test item, compared to 10% in the voluntary group, with a Chi-square (1, 265) = 7.15, *p* = 0.004. Furthermore, 46% of participants in the mandatory group scored 0, while 1.3% scored 10 on all items. In the voluntary group, the corresponding figures were 26.7% and 0%, respectively, with a Chi-square (1, 265) = 4.55, *p* = 0.025. The results also showed that 87.1% of participants in the mandatory group had at least one gambling day post-intervention, compared to 86.0% in the voluntary group and 85.2% in the control group (Chi-square (2, 1825) = 0.932, *p* = 0.627). These findings supported hypotheses H1 (engagement), H2 (quality), and H5 (account attrition). Since H5 was a null hypothesis and therefore more susceptible to insufficient power, this analysis was re-run using a Bayesian equivalent contingency table. Findings from these Bayesian post-hoc analyses (run with a standard, weakly informative prior concentration of 1) revealed that the null hypothesis was indeed more probable: the BF_01_ value was 293.42 when running the full 3 × 2 model, and in all pairwise contrasts, BF_01_ > 12.63.

On the other hand, there were no differences observed in terms of clicks on limits (Chi-square (2, 1825) = 2.74, *p* = 0.254), self-exclusions (Chi-square (2, 1825) = 2.69, *p* = 0.261), or clicks on gambling history (Chi-square (2, 1825) = 3.93, *p* = 0.14). Additionally, as shown in Table 1, there were no significant differences between the groups regarding time spent, bets placed, or net losses. However, a higher proportion of participants in the mandatory group (6.1%) clicked on the Normative test “How do you compare,” compared to 1.8% in the voluntary group and 0.5% in the control group (Chi-square (2, 1825) = 38.7, *p* < 0.001). As such, H4 (use of RG tools) was partially supported, while H3 (impact on subsequent gambling) was not.

**Table 1:** Repeated measures GLM for theoretical loss (TL), net expenditure, days played and minutes played

Variables		Group Mean (SD) in SEK, days, and minutes respectively			GLM results	
		Control n = 600	Voluntary n = 621	Mandatory n = 604	Time	Time*Group
TL	Pre	623(1888) 309 (794)	568 (1597) 376 (2765)	758 (2855) 315 (1471)	F(1) = 31.9 P < .001	F(2) = 1.68 P = .186
	Post					
Net expenditure*	Pre	919(1870) 591(1476)	719(2331) 376(1061)	919 (2426) 560(1580)	F(1) = 38.6 P < .001	F(2) = .031 P = .970
	Post					
Days played	Pre	7.3 (5.7) 5.8 (6.0)	7.2 (5.6) 5.6 (5.8)	7.4 (5.9) 5.8 (6.2)	F(1) = 132 P < .001	F(2) = .061 P = .768
	Post					
Minutes	Pre	273 (633) 241 (706)	205 (444) 197 (637)	274 (720) 236 (634)	F(1) = 4.63 P < .001	F(2) = .574 P = .563
	Post					

^*^excluding winners > 1000 pre & post, n = 1326

## Discussion

The use of self-test tools by gambling operators has increased around the world, driven by their ease of integration into online gambling platforms and expanding duty of care obligations that include evaluating risky gambling behavior. Current research [32–34] suggests that ‘intrusive’ duty of care measures, such as mandatory self-assessment tools, motivational phone calls, or strict universal budget limits, are generally well-received by gambling customers and do not affect players’ loyalty. As a result, the implementation of self-test tools is expected to continue, being widely adopted by gambling operators as both preventive and curative tools including facilitating informed choices, engaging players with responsible gambling themes, and assessing players’ risk. Most operator-based self-test implementations, however, continue to rely on voluntary participation and have faced criticism for their limited positive effect on gambling behavior [35, 36].

This study sought to enhance our understanding of the factors contributing to the effectiveness of self-test tools by examining how different formats (mandatory vs. voluntary) influence adoption, response quality, time spent on the test, and subsequent gambling behavior. In addition, we’ve evaluated the impact of self-test presentations on customer retention and continued use of gambling services. Our findings indicate that while mandatory self-tests lead to (much) higher participation, they may not effectively achieve all the primary goals of self-tests for all customers, such as assessing risk, promoting informed decision-making, or encouraging healthier gambling behavior. The results also indicate that voluntary self-assessment tools can lead to somewhat better test quality outcomes, though this may not necessarily translate into healthier gambling behavior. Additionally, our study replicates the current consensus that self-test tools do not harm operator-customer relationships or negatively impact customer loyalty, operationalized as churn. The results showed no effect on gambling behavior, which partly can be explained by the fact that only 3% in the intervention groups performed at-risk or problematic self-tests. A great majority of those taking a self-test got feedback that they showed no signs of problems, which calls for a status quo rather than change.

Consequently, the success of self-assessment tools as a duty of care measure depends on the balance between achieving high participation and maintaining user engagement. Findings of the current study suggest that while ensuring widespread use, mandatory tests may also result in some users rushing through them without genuine engagement, reducing the quality of responses and the test’s effectiveness. Additionally, some users might find ways to bypass mandatory tests which can lead to a false sense of effective screening and reporting among the operators. Indeed, previous studies [37, 38] indicate that the effectiveness of mandatory measures in addiction settings can be undermined by a lack of motivation, relevance, and noncompliance in the form of circumventive requirements. Thus, superficial compliance aimed at completing self-tests, perceptions of paternalism, and a lack of relevance to individual gambling habits and personal benefits may contribute to resistance and poorer quality of mandatory self-tests. Further research is needed to investigate these factors and promote engagement by encouraging personal reflection, enhancing relevance, and supporting user autonomy.

Second, the result also reveals that voluntary self-tests, although less effective in attracting widespread use, may have a better appeal to players’ engagement. As the pop-up self-test invitation clearly shows a way to cancel taking the test, the test invitation design alleviates the pressure of taking it—which can appeal to players’ sense of independence. In fact, such compliance design is one of the central principles of user-centered design [39] in that ‘choice autonomy can increase recommendation acceptance’ and foster ownership and honest reflection Since the self-test was presented as optional, participants could engage with it at their own discretion and when they felt ready—which may suggest timing may play a factor. Moreover, offering multiple opportunities inherently creates a subtle nudging effect while preserving the autonomy of the decision. This approach minimizes direct pressure, encouraging those who choose to take the test to do so with genuine reflection and honesty. Further research is needed to evaluate these hypotheses.

## Implications

Understanding the implementation format of self-assessment tools is essential to their effectiveness, whether they are used in different settings—such as supporting informed decision-making or screening—or for various objectives, such as prevention or intervention. Our findings suggest that the presentation format—whether as a mandatory or voluntary test—can influence user perception, engagement, and the overall quality of use. Since these tools are primarily implemented in technological settings (e.g., online platforms), the use of self-test tools as RG-measures is as much a matter of gambling knowledge base as it is of technological and behavioral design. In line with other researchers [40] who have advocated for nuanced strategies in implementing these tools, the field should place greater emphasis on human-centered design principles, cognitive bias behavior, and decision-making processes to achieve overall quality of use.

The results also have practical implications for policymaking in the context of the duty of care and operators’ agenda of RG measures. First, gambling operators should exercise caution when interpreting non-risk assessment results derived solely from self-test tools, particularly when these tools are implemented in a mandatory format, as it can result in inaccurate risk assessment. This said, the mandatory setting collected far more tests with good quality than the voluntary format in absolute numbers. A more nuanced approach can be a combination of quality checked self-tests together with real-time behavioral data such as betting and withdrawal behaviors. Second, over-reliance on self-tests as the default risk-assessment method to meet the duty of care obligations can be misleading, as it may lead to superficial compliance by operators in fulfilling their responsibilities. Policymakers may need to offer more detailed guidance on the implementation of such tools to ensure operators effectively achieve their risk assessment obligation. Moreover, self-test tool developers within gambling operators (e.g., UX and data science teams) need to adopt a more socio-technical approach that integrates human-centered design principles. Such an approach might involve experimenting with different presentations of the self-test tool (e.g., A/B testing) to determine the most effective format for optimal use of these measures.

Finally, the limited effectiveness of self-assessment tools underscores that self-management and personal responsibility may not always be sufficient to promote healthy gambling behaviors. As previously mentioned, measures that are less influenced by individual choices can offer a reliable approach to implement harm minimization measures, including regulating gambling product design, marketing restrictions, or supervised self-assessment tools [41, 42]. That said, our null hypothesis findings can serve as a foundation for further research exploration, including comparisons between self-reliant tools and supervised interventions that are more stringent harm-minimization strategies to address problem gambling.

### Strength, limitations, and future research

The current research has several limitations that should be considered. First, as the data comes from a single operator, further studies using diverse datasets are warranted to replicate the findings across various contexts. However, it should be noted that operator in question is among the largest in Sweden and is likely top-of-mind among large proportions of the gambling-interested Swedish population. Second, the respondents were sampled from a limited time frame—pre- and post-gambling periods of just four weeks each—to assess the effect of self-test methods on player engagement. This relatively short duration may not fully capture long-term effects, and further research with longer observation periods could provide a more comprehensive understanding of the impact of self-test tools on user behavior over time. Alternatively, immediate and transient short-term effects may have been obfuscated when collapsing data into a four-week post period; however, many (problem) gamblers display a periodic, high-intensity gambling behavior (typically around payday, when money is available) that requires a longer follow-up duration to capture. Moreover, one could argue that any effects on gambling behavior must be prolonged and stable enough to be detectable with a sample size of this magnitude, also over a four week period. Third, participants in this study were drawn from a new customer pool, which represents a specific population cohort with a potential pattern of engagement. As such, future research should examine self-test presentation methods with a broader participant base that includes both new and experienced users from different operators in a longitudinal setting. Another limitation is that the mandatory condition was not truly mandatory: it was technically possible to circumvent, which a majority did. Finally, statistical power may have been insufficient: a posthoc power analysis revealed that the sample at hand could detect (with 80% power) a post-intervention pairwise difference of d > 0.16 with the observed SD to mean ratios (i.e. coefficients of variation) of around 2.5, rendering analyses on some of the numeric outcomes potentially underpowered. In comparing churn, power was only 14.7% when comparing the observed maximum difference of 87.1% re-engagement against the 85,2% observed in the control group; here, a re-engagement of 90.1% would have been required to achieve 80% power. While we cannot rule out that a larger sample would have resulted in significant findings, the magnitude of the effect is itself of public health importance: effects requiring a sample size in the hundreds of thousands would arguably have limit policy implications since the public health impact would be near negligible, making other measures more attractive.

To the best of our knowledge, the present study is the first to exclusively compare mandatory and voluntary gambling self-test delivery methods in the context of their influence on user engagement as well as their impact on the use of other RG tools. Additionally, the study’s experimental design enhances its ecological validity, as it mirrors real-world conditions in which individuals interact with gambling tools, providing a robust foundation for future research in this area. Finally, since participants were selected from new customers, the findings may offer a glimpse into initial levels of user engagement and the quality of self-assessment tools as preventive measures.

## Conclusion

The growing availability of gambling on online platforms, coupled with regulation-mandated duty of care measures, ensures that self-test assessment approaches will continue to be a key tool in the field for the foreseeable future. The overall ambition of the current study was to add knowledge to the use and effects of gambling self-tests as an RG measure. We compared a mandatory (but avoidable) invitation to take a self-test with receiving multiple reminders, and controls. The mandatory invitation resulted in more performed self-tests, with a somewhat lower quality, and there was no difference in customer survival. There was no effect on subsequent gambling behavior and a limited effect on use of RG measures. Further research is needed to explore how to implement self-tests in the gambling companies’ duty-of-care in an optimal way.

## Data Availability

Due to the nature of the research, due to commercial secrecy, supporting data is not available.

## References

[1] Marionneau V, Järvinen-Tassopoulos J. Consumer protection in licensed online gambling markets in France: the role of responsible gambling tools. Addict Res Theory. 2017;25(6):436–43.

[2] Meerkerk G-J. Gambling legislation on duty of care and limit setting in 22 European countries. Dutch Gambling Authority. 2022

[3] Kelly JM, Igelman A. Compulsive gambling litigation: casinos and the duty of care. Gaming Law Rev Econ. 2009;13(5):386–403.

[4] Motka F, Gruene B, Sleczka P, Braun B, Örnberg JC, Kraus L. Who uses self-exclusion to regulate problem gambling? A systematic literature review. J Behav Addict. 2018;7(4):903–16. 30378459 10.1556/2006.7.2018.96 PMC6376385

[5] Blank L, Baxter S, Woods HB, Goyder E. Should screening for risk of gambling-related harm be undertaken in health, care and support settings? A systematic review of the international evidence. Addict Sci Clin Pract. 2021;16(1):35. 34051852 10.1186/s13722-021-00243-9 PMC8164740

[6] Keen B, Blaszczynski A, Anjoul F. Systematic review of empirically evaluated school-based gambling education programs. J Gambl Stud. 2017;33:301–25. 27566689 10.1007/s10899-016-9641-7

[7] Nower L, Glynn J. Adopting an affordability approach to responsible gambling and harm reduction: considerations for implementation in a North American Context. Gaming Law Rev Econ Regul Compliance Policy. 2022;26(9):466–76. 10.1089/glr2.2022.0020.

[8] Stinchfield R, McCready J, Turner N. A comprehensive review of problem gambling screens and scales for online self-assessment. Toronto: Ontario Problem Gambling Research Centre; 2012.

[9] Otto JL, Smolenski DJ, Wilson ALG, Evatt DP, Campbell MS, Beech EH, Workman DE, Morgan RL, O’Gallagher K, Belsher BE. A systematic review evaluating screening instruments for gambling disorder finds lack of adequate evidence. J Clin Epidemiol. 2020;120:86–93. 31917356 10.1016/j.jclinepi.2019.12.022

[10] Volberg RA, Williams RJ. Developing a brief problem gambling screen using clinically validated samples of at-risk, problem and pathological gamblers. Health Sciences. 2011

[11] Volberg RA, Munck IM, Petry NM. A quick and simple screening method for pathological and problem gamblers in addiction programs and practices. Am J Addict. 2011;20(3):220–7. 21477050 10.1111/j.1521-0391.2011.00118.x PMC3076109

[12] Goodyear-Smith F, Martel R, Darragh M, Warren J, Thabrew H, Clark TC. Screening for risky behaviour and mental health in young people: the YouthCHAT programme. Public Health Rev. 2017;38:1–12. 29450092 10.1186/s40985-017-0068-1 PMC5810064

[13] Hodgins DC, Cunningham JA, Murray R, Hagopian S. Online self-directed interventions for gambling disorder: randomized controlled trial. J Gambl Stud. 2019;35:635–51. 30701377 10.1007/s10899-019-09830-7

[14] Sullivan S. Don’t let an opportunity go by: validation of the EIGHT gambling screen. Int J Ment Heal Addict. 2007;5:381–9.

[15] Jonsson J, Hodgins DC, Lyckberg A, Currie S, Young MM, Pallesen S, Carlbring P. In search of lower risk gambling levels using behavioral data from a gambling monopolist. J Behav Addict. 2022;11(3):890–9. 10.1556/2006.2022.00062. 36125925 10.1556/2006.2022.00062 PMC9872526

[16] Adams PJ, Rossen F. A tale of missed opportunities: pursuit of a public health approach to gambling in New Zealand. Addiction. 2012;107(6):1051–6. 10.1111/j.1360-0443.2012.03800.x. 22563834 10.1111/j.1360-0443.2012.03800.x

[17] Van Schalkwyk MCI, Petticrew M, Cassidy R, Adams P, McKee M, Reynolds J, Orford J. A public health approach to gambling regulation: countering powerful influences. Lancet Public Health. 2021. 10.1016/S2468-2667(21)00098-0. 34166631 10.1016/S2468-2667(21)00098-0

[18] Livingstone C, Rintoul A. Moving on from responsible gambling: a new discourse is needed to prevent and minimise harm from gambling. Public Health. 2020;184:107–12. 10.1016/j.puhe.2020.03.018. 32434694 10.1016/j.puhe.2020.03.018

[19] Selin J. Politics of hesitance and the formation of ethical subjects through responsible gambling practices. Front Sociol. 2022;7:1032752. 36589790 10.3389/fsoc.2022.1032752 PMC9795170

[20] Shaffer HJ, Blaszczynski A, Ladouceur R. Gambling control and public health: let’s be honest. Int J Ment Heal Addict. 2020;18:819–24.

[21] Marko S, Thomas SL, Robinson K, Daube M. Gamblers’ perceptions of responsibility for gambling harm: a critical qualitative inquiry. BMC Public Health. 2022;22(1):725. 10.1186/s12889-022-13109-9. 35413823 10.1186/s12889-022-13109-9 PMC9004097

[22] Carneiro E, Tavares H, Sanches M, Pinsky I, Caetano R, Zaleski M, Laranjeira R. Gender differences in gambling exposure and at-risk gambling behavior. J Gambl Stud. 2020;36:445–57. 31471835 10.1007/s10899-019-09884-7

[23] Forsström D, Jansson-Fröjmark M, Hesser H, Carlbring P. Experiences of Playscan: interviews with users of a responsible gambling tool. Internet Interv. 2017;8:53–62. 30135829 10.1016/j.invent.2017.03.003 PMC6096211

[24] Langenbucher J, Bavly L, Labouvie E, Sanjuan PM, Martin CS. Clinical features of pathological gambling in an addictions treatment cohort. Psychol Addict Behav. 2001;15(1):77. 11255942 10.1037/0893-164x.15.1.77

[25] Rodda S, Lubman DI. Preoccupation, gambling and the DSM-V. Int Gambling Stud. 2012;12(3):421–2. 10.1080/14459795.2012.698296.

[26] Hare, S. Study of gambling and health in Victoria: findings from the Victorian Prevalence Study 2014, Victorian Responsible Gambling Foundation and Victorian Department of Justice and Regulation, Melbourne. 2015.

[27] Woods A, Sproston K, Brook K, Delfabbro P, O’Neil M. Gambling prevalence in south Australia (2018). 2019

[28] Wardle H. British gambling prevalence survey 2007. The Stationery Office. 2007

[29] Jonsson J, Munck I, Volberg R, Carlbring P. GamTest: psychometric evaluation and the role of emotions in an online self-test for gambling behavior. J Gambl Stud. 2017;33:505–23. 28265831 10.1007/s10899-017-9676-4 PMC5445150

[30] Jonsson J. Internet poker in Sweden in 2007. In Routledge international handbook of internet gambling. Routledge; 2012. p. 126–139

[31] Delfabbro PH, King DL. The value of voluntary vs. mandatory responsible gambling limit-setting systems: a review of the evidence. Int Gambl Stud. 2021;21(2):255–71. 10.1080/14459795.2020.1853196.

[32] Ivanova E, Rafi J, Lindner P, Carlbring P. Experiences of responsible gambling tools among non-problem gamblers: a survey of active customers of an online gambling platform. Addict Behav Rep. 2019;9:100161. 10.1016/j.abrep.2019.100161. 31193727 10.1016/j.abrep.2019.100161 PMC6542737

[33] Jonsson J, Hodgins DC, Munck I, Carlbring P. Reaching out to big losers: a randomized controlled trial of brief motivational contact providing gambling expenditure feedback. Psychol Addict Behav. 2019;33(3):179–89. 10.1037/adb0000447. 30829516 10.1037/adb0000447

[34] Lakew N. “Show me the money”: Preliminary lessons from an implementation of intervention tools at the payment gateway level. J Gambl Stud. 2022;38(1):297–317. 10.1007/s10899-021-10023-4. 33761066 10.1007/s10899-021-10023-4

[35] Abbott MW. Commentary on Currie et al. (2017) Low-risk gambling limits-a bridge too far? Addiction. 2017;112(11):2021–2. 10.1111/add.14017. 28990302 10.1111/add.14017

[36] Tanner J, Drawson AS, Mushquash CJ, Mushquash AR, Mazmanian D. Harm reduction in gambling: a systematic review of industry strategies. Addict Res Theory. 2017;25(6):485–94. 10.1080/16066359.2017.1310204.

[37] Gainsbury SM, Angus DJ, Procter L, Blaszczynski A. Use of consumer protection tools on internet gambling sites: customer perceptions, motivators, and barriers to use. J Gambl Stud. 2020;36(1):259–76. 10.1007/s10899-019-09859-8. 31119509 10.1007/s10899-019-09859-8

[38] Long BR. Theorising gambling self-exclusion agreements: the inadequacy of procedural autonomy. Can J Law Jurisprud. 2023. 10.1017/cjlj.2022.30.

[39] Fink L, Newman L, Haran U. Let me decide: increasing user autonomy increases recommendation acceptance. Comput Hum Behav. 2024;156:108244.

[40] Forsstrom D, Rafi J, Carlbring P. Dropouts’ usage of a responsible gambling tool and subsequent gambling patterns. Cogent Psychol. 2020. 10.1080/23311908.2020.1715535.

[41] McGrane E, Wardle H, Clowes M, Blank L, Pryce R, Field M, Sharpe C, Goyder E. What is the evidence that advertising policies could have an impact on gambling-related harms? A systematic umbrella review of the literature. Public Health. 2023;215:124–30. 10.1016/j.puhe.2022.11.0192. 36725155 10.1016/j.puhe.2022.11.019

[42] Newall PWS. Reduce the speed and ease of online gambling in order to prevent harm. Addiction. 2022;24:24. 10.1111/add.16028. 10.1111/add.16028 36002979

